# Systemic Injection of Low-Dose Lipopolysaccharide Fails to Break down the Blood–Brain Barrier or Activate the TLR4-MyD88 Pathway in Neonatal Rat Brain

**DOI:** 10.3390/ijms150610101

**Published:** 2014-06-05

**Authors:** Peng Wang, Si-Wei You, Yin-Jie Yang, Xiao-Yan Wei, Ya-Zhou Wang, Xin Wang, Ding-Jun Hao, Fang Kuang, Li-Xin Shang

**Affiliations:** 1 Institute of Neurosciences, the Fourth Military Medical University, 169 Changle West Road, Xi’an 710032, China; E-Mails: wpfmmu@163.com (P.W.); yousiwei@fmmu.edu.cn (S.-W.Y.); xiaoywei@fmmu.edu.cn (X.-Y.W.); yazhouw@fmmu.edu.cn (Y.-Z.W.); 2 Department of Obstetrics and Gynecology, General Hospital of Beijing Military Region, 5 Nanmencang Road, Beijing 100700, China; E-Mail: wangxinbzfc@163.com; 3 Department of Neurology, the 425th People’s Liberation Army Hospital, 86 Sanyawan Road, Sanya 572000, China; E-Mail: yangyinj@163.com; 4 Department of Spine Surgery, Xi’an Red Cross Hospital, 555 Youyi East Road, Xi’an 710054, China

**Keywords:** low-dose lipopolysaccharide, blood–brain barrier, toll-like receptor 4, neonatal, rat

## Abstract

We aimed to investigate whether peripheral low-dose lipopolysaccharide (LPS) induces the breakdown of the blood–brain barrier (BBB) and/or the activation of toll-like receptor 4 (TLR4) in the neonatal rat brain. Neonatal rats received intraperitoneal injections of low-dose LPS (0.3 mg/kg∙bw), and the BBB compromise was detected by Evans Blue extravasation and electron microscopy. Meanwhile, TLR4, adaptin myeloid differentiation factor 88 (MyD88), nuclear transcription factor kappa-B (NF-κB) p50 and tumor necrosis factor alpha (TNFα) in the neonatal rat brain were determined by quantitative real-time polymerase chain reaction (PCR) and Western Blot. Immunohistochemistry was used to determine the distribution and activation of microglia in the brain after LPS administration. It was demonstrated that Evans Blue extravasation was not observed in the brain parenchyma, and that tight junctions of cerebral endothelial cells remained intact after systemic injections of LPS in neonatal rats. Although intracerebroventricular injections of LPS activated microglia and up-regulated the expression of TLR4, MyD88, NF-κB p50 and TNFα in the neonatal rat brain, systemic LPS did not induce these responses. These findings indicate that while the neonatal rat brain responds to the direct intra-cerebral administration of LPS through robust TLR4 activation, systemic low-dose LPS does not induce the innate immune reaction or compromise the BBB in neonatal rats.

## 1. Introduction

As a component of gram-negative bacteria, lipopolysaccharide (LPS) is able to induce immune responses in both the peripheral and central nervous system (CNS) of adults [1,2], causing diverse reactions upon entry into the organism [3]. As peripheral LPS may induce an immune reaction in the CNS via several presumed pathways [4,5,6,7,8], systemic injection of LPS is critical for the initiation of the inflammatory reaction in the developing CNS of neonates. In comparison with a high dose of LPS infection that causes sepsis or peritonitis, a low level of LPS in circulating blood is detected more commonly in a number of non-lethal conditions and diseases. However, whether low-dose LPS in circulating blood affects the CNS immune reaction of neonates is still unclear.

The blood–brain barrier (BBB) is mainly composed of brain capillary endothelial cells, pericytes and astrocyte endfeet [9,10], and plays a key role in the maintenance of CNS homeostasis. Under normal conditions, large molecules including LPS cannot pass through the BBB [11,12]. Under certain pathological conditions such as systemic inflammation, cerebral ischemia and subsequent reperfusion, brain tumors, trauma and diabetes, BBB permeability increases [13]. Notably, the immature BBB seems more vulnerable to proteins and some large molecules than the mature BBB in adults [14]. Thus, the immature BBB might be challenged by peripheral LPS; if the immature BBB is compromised upon the systemic injection of LPS, circulating proteins, including plasma proteins and cytokines, and other damage associated molecular patterns (DAMPs) are allowed to enter the brain and activate toll-like receptor 4 (TLR4), a specific ligand for LPS and other DAMPs [15,16]. The intracellular adaptor protein myeloid differentiation factor 88 (MyD88)-dependent pathway will be subsequently activated, resulting in the production of cytokines via the translocation of nuclear transcription factor kappa-B (NF-κB) into the nucleus [17]. Some recent studies have shown that the systemic injection of low-dose LPS protected the brain against hypoxia ischemic (HI) injury, and that the neuroprotective effect of LPS preconditioning was induced by the activation of the innate immune response pathways in the brain [14,18,19,20]. In these studies, the mechanisms of LPS preconditioning are still controversial. Bordet *et al.* found that the neuroprotective effect of low-dose LPS was mediated by an increased synthesis of brain superoxide dismutase (SOD) that was triggered by activation of the inflammatory pathway [21]. Ikeda *et al.* indicated that the up-regulation of endogenous corticosterone appeared to participate in LPS-induced cerebral tolerance in neonatal rats [22]. Other studies demonstrated that the peripheral administration of LPS induced the expression of proinflammatory cytokines including tumor necrosis factor-α (TNF-α), interleukin-6 (IL-6) and interleukin-1β (IL-1β) in the brain [23,24,25]. LPS also induced up-regulation of ceramide which is a downstream messenger in TNF-α signaling in the plasma and brain cortex [26]. All these reports suggest that low-dose LPS triggers slight inflammation that causes HI tolerance. In neonatal rats, preconditioning with low-dose LPS seems to be dependent on their development. For instance, preconditioning with low-dose LPS (0.1 mg/kg·bw) for 48 h before ischemia reduced brain damage in postnatal day 7 (P7), P9 and P14 rat pups, but not in P3 and P5 rats [20]. Whether the CNS of younger neonates responds to systemic LPS via a TLR4-mediated innate immune reaction is still unknown.

In order to investigate whether the BBB and central TLR4-mediated innate immune reaction can be affected by peripheral low-dose LPS in the neonate, we studied BBB integrity and TLR4 expression in neonatal rats after intraperitoneal (ip.) injection (*i.e.*, systemic or peripheral injection) of low-dose LPS. Intracerebroventricular (icv.) injection (*i.e.*, central injection) of LPS was also used to investigate the central TLR4 response. Surprisingly, systemic injection of low-dose LPS led to neither BBB compromise nor TLR4 up-regulation in the brain, while the immature brain responded to the central injection of LPS with significantly increased TLR4 expression and activation of the downstream signaling pathway.

## 2. Results and Discussion

### 2.1. Unchanged Blood–Brain Barrier (BBB) after Systemic Lipopolysaccharide (LPS)

#### 2.1.1. Evans Blue (EB) Extravasation

**Figure 1:**
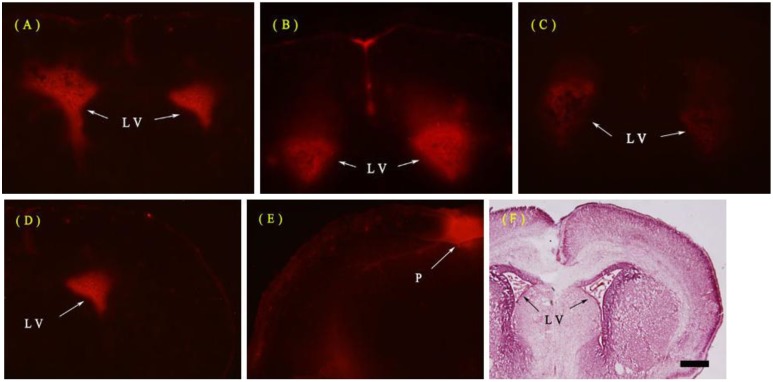
Representative photographs for Evans Blue (EB) extra-vasation in the neonatal rat brain at postnatal day 1 (P1). (**A**–**C**) shows no red fluorescence of EB in all the levels of the rat brain 6, 12 or 24 h after intraperitoneal (ip.) injection of lipopolysaccharide (LPS) except for the lateral ventricle (LV) choroid plexus (**the arrows**), compared with normal saline (NS) group; (**D**–**E**) shows significant EB staining in the pineal body (P) (**the arrow**); (**F**) shows the HE staining structure of the P1 rat brain at the same coronal section. Bar for **A**–**E**, 50 µm.

The whole brain was white when removed except for non-BBB areas such as the pineal bodies and pituitary. These non-BBB structures were stained blue in both LPS and normal saline (NS) control groups. Fluorescent microscopy showed no red fluorescence of Evans Blue (EB) in the parenchyma of the neonatal rat brain at 6, 12 or 24 h after ip. injection of LPS at all ages (postnatal days 3 and 7 (P3 and P7) were not shown) except for non-BBB areas, such as the pineal bodies, subfornical organs, choroid plexus and pial mater in both ip. LPS and NS groups (Figure 1).

#### 2.1.2. Fine Structure of the BBB

**Figure 2:**
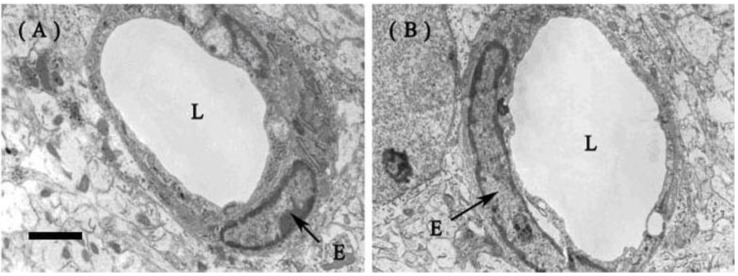
Representative photographs of transmission electron microscopy for the neonatal rat brain at P1. (**A**) The capillaries remained normal and the endothelial cells and tight junctions were intact in the parenchyma of the rat brain (frontal cortex) 12 h after ip. injection of LPS; (**B**) The brain (frontal cortex) of the control rat 12 h after ip. injection of NS. (L, luminal surface; E, endothelial cell). Bar for **A** and **B**, 1 µm.

Transmission electron microscopy showed basically normal fine structures of the cerebral endothelia and other neural cells in both LPS and NS groups. There was no significant alteration of endothelial tight junction 12 h after exposure to ip. LPS in the developing brains (Figure 2). No abnormality was observed in other parenchymal areas away from the capillaries.

### 2.2. No Toll-Like Receptor 4 (TLR4) Protein and mRNA Responses to Peripheral LPS

**Figure 3:**
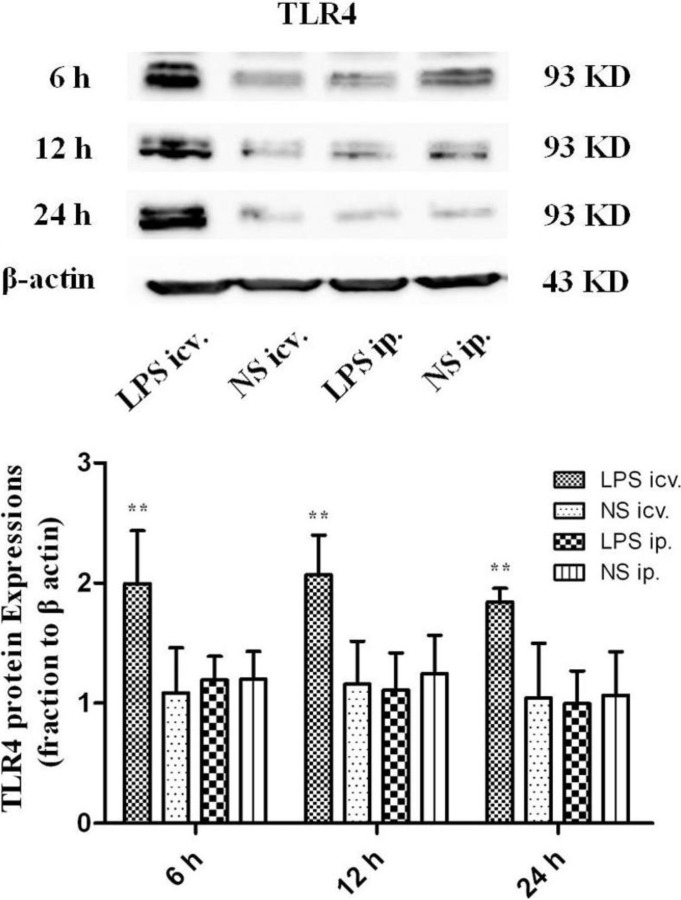
The protein levels ofToll-like receptor 4 (TLR4) in the P1 rat brain 6, 12 and 24 h after intracerebroventricular (icv.) or ip. injections of LPS or NS. At all time points detected, icv. injection of LPS induced dramatic increases of TLR4 protein expression (** *p* < 0.01), compared with those in the peripheral LPS, central NS and peripheral NS injection groups. No significant difference was found in the expression of TLR4 protein in the neonatal rat brains between ip. injections of LPS and NS.

Western Blot showed that the protein levels of TLR4 were all significantly up-regulated (*p* < 0.01) in the P1 rat brain 6, 12 and 24 h after icv. injections of LPS compared with those in the peripheral LPS, central NS and peripheral NS injection groups. However, no such significant differences were detected in the P1 rat brain between the ip. LPS and NS groups at all time points (Figure 3).

**Figure 4:**
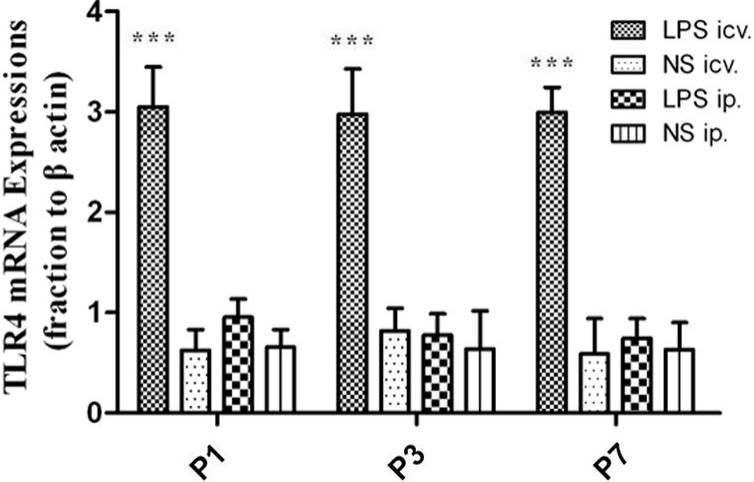
The expression of TLR4 mRNA in the neonatal rat brain. At all ages detected, icv. injection of LPS induced dramatic increase in TLR4 mRNA expression (*** *p* < 0.001), compared with that in the peripheral LPS, central NS and peripheral NS injection groups. No significant difference was found in the TLR4 mRNA expression in the neonatal rat brains between ip. injections of LPS and NS.

Quantitative real-time polymerase chain reaction (PCR) indicated that the expression level of TLR4 mRNA was significantly increased (*p* < 0.001) in the neonatal rat (P1, P3 and P7) brain treated with icv. injections of LPS, compared with that in the peripheral LPS, central NS and peripheral NS injection groups. However, there was no significant difference in the expression of TLR4 mRNA between LPS and NS groups 12 h after ip. injections of LPS or NS in all the neonatal brains (Figure 4). The amplification of each batch was repeated three times.

### 2.3. Myeloid Differentiation Factor 88 (MyD88)–Nuclear Transcription Factor Kappa-B (MyD88–NF-κB) Pathway Is not Activated by Systemic LPS

**Figure 5:**
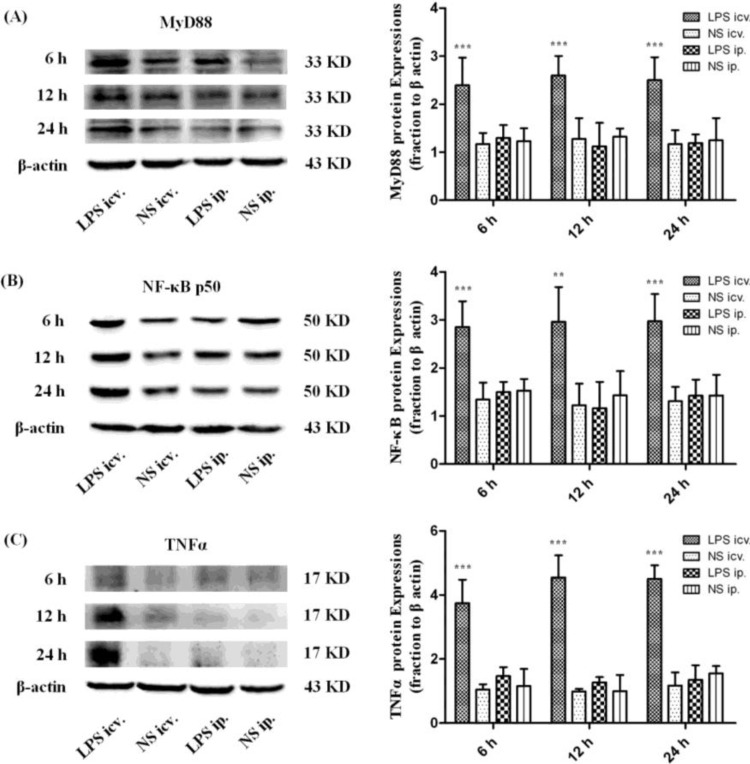
The protein levels of myeloid differentiation factor 88 (MyD88), nuclear transcription factor kappa-B (NF-κB) p50 and tumor necrosis factor alpha (TNFα) 6, 12 and 24 h after icv. or ip. injection of LPS or NS in the P1 rat brain. (**A**) Representative picture of the immunoblot of MyD88 in the P1 rat brain after icv. or ip. injection of LPS or NS at 3 time points, and the graph of statistical analysis of the immunoblot of MyD88 in the brain in various groups. *** *p* < 0.001, compared with the central NS injection group; (**B**) Representative picture of the immunoblot of NF-κB p50 in the P1 rat brain after icv. or ip. injection of LPS or NS at 3 time points, and the graph of statistical analysis of the immunoblot of NF-κB p50 in the brain in various groups. ** *p* < 0.01 and *** *p* < 0.001, compared with the central NS injection group; (**C**) Representative picture of the immunoblot of TNFα in the P1 rat brain after icv. or ip. injection of LPS or NS at 3 time points, and the graph of statistical analysis of the immunoblot of TNFα in the brain in various groups. *** *p* < 0.001, compared with the central NS injection group.

**Figure 6:**
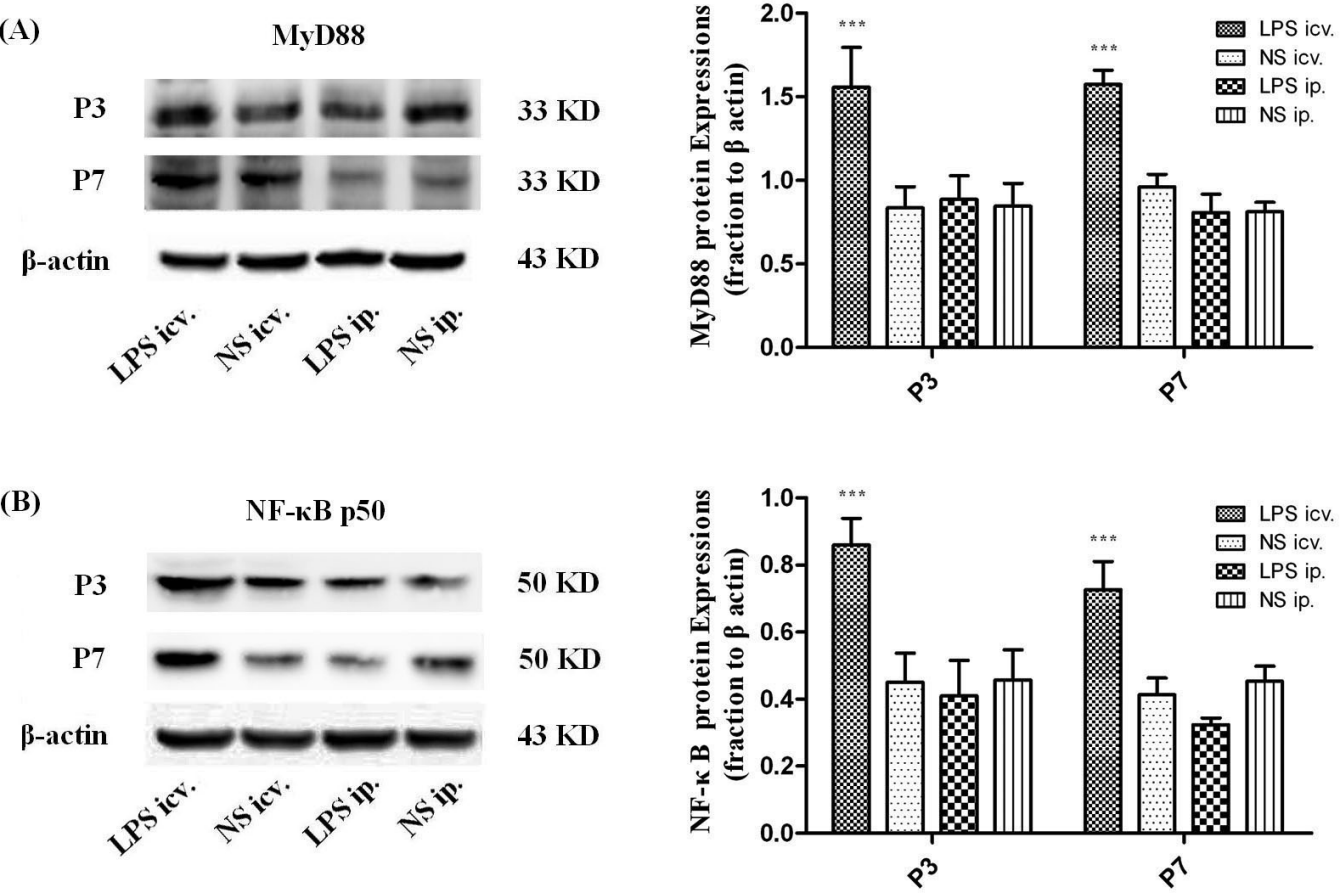
The protein levels of MyD88 and NF-κB p50 in the neonatal rat (P3 and P7) brains 12 h after icv. or ip. injection of LPS or NS. (**A**) Representative picture of the immunoblot of MyD88 in the neonatal rat brains treated with the central or peripheral LPS or NS, and the graph of statistical analysis of the immunoblot of MyD88 in the brain in various groups. *** *p* < 0.001, compared with the central NS injection; (**B**) Representative picture of the immunoblot of NF-κB p50 in the neonatal rat brain treated with the central or peripheral LPS or NS, and the graph of statistical analysis of the immunoblot of NF-κB p50 in the brain in various groups. *** *p* < 0.001, compared with the central NS injection.

Western Blot assay showed that the protein levels of MyD88, NF-κB p50 and TNFα were all significantly up-regulated in the P1 rat brain 6, 12 and 24 h after icv. injection of LPS compared with those in peripheral LPS, central NS and peripheral NS injection groups. However, no such significant differences could be detected in the P1 rat brain between the peripheral LPS and NS injection groups at all time points (Figure 5). Similar results were obtained in the expressions of MyD88 and NF-κB p50 in the neonatal rat brain (P3 and P7) 12 h after icv. or ip. injection of LPS or NS (Figure 6).

### 2.4. Microglia Is not Activated after Systemic LPS in Neonatal Rat Brain

**Figure 7:**
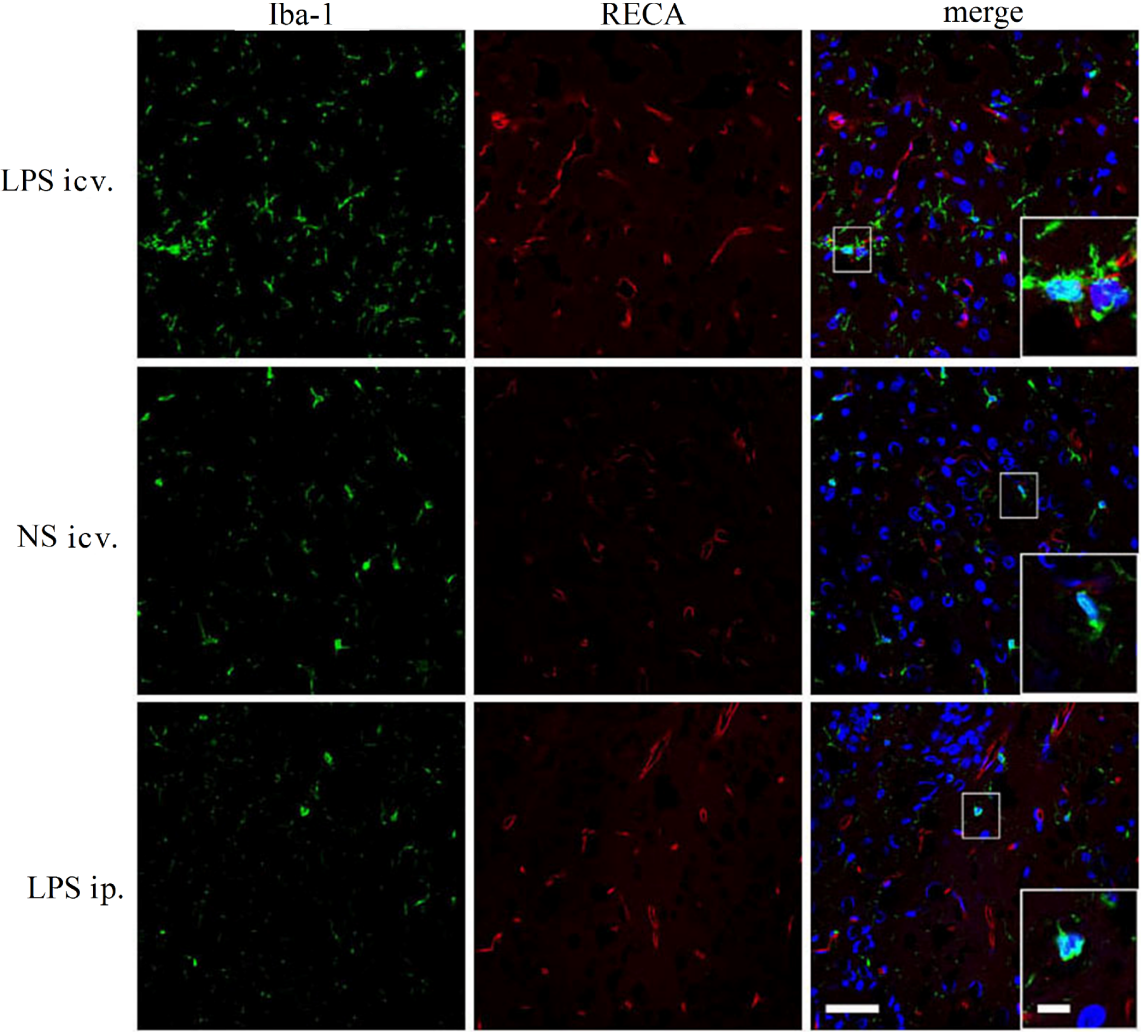
Representative micrographs of immunofluorescent labeling for Iba-1 (green) and RECA (red) in the brains of the rat at P1. In the central LPS injection group, Iba-1 positively labeled microglial cells distributed more widely than that in the brain (frontal cortex in this figure) treated with ip. injection of LPS or NS. Microglial cells were activated (indicated by large cell bodies and thick processes) while in the brains of rats treated with ip. injectionsof LPS, there were fewer Iba-1 positively labeled cells and these cells remained in the resting state (indicated by their cell bodies and processes), similar to those in the NS-treated brain. RECA immunolabeling revealed no significant difference in the morphology and distribution of the capillaries among these three groups. Amplified boxes were inserted in the merged pictures. Bar outside the box for all the images, 50 µm; Bar inside the box for all the insertions, 10 µm.

Double immunolabeling for Iba-1 with RECA clearly presented the morphology of the microglia and the distribution of the microvessel in the neonatal rat brain. Microglia was strongly activated 12 h after icv. injection of LPS in neonatal rat brain (P1, P3 and P7). Although the activated microglial cells were distributed in all areas of the brain, there was no tendency of any correlation between the activation of the microglia and the distribution of the capillaries. However, no microglial activation was found in the central LPS and NS injection groups (Figure 7).

### 2.5. Discussion

Peripherally administered LPS may affect the CNS, but the circulating LPS is unlikely to enter the CNS and invoke neuroimmune events unless the highest dose of LPS is used for the most sensitive brain [12]. A low dose of peripheral LPS significantly induced the increase of plasma proinflammatory cytokines including IL-6 12–24 h after injection in the adult rats [26]. These LPS-induced cytokines were deduced to compromise either the structure or the function of the BBB [27]. However, data in the present study showed that low-dose LPS injected peripherally did not break down the BBB of the neonatal rats.

EB is widely used as a BBB tracer because it binds serum albumin almost entirely and immediately after entering the blood [28]. So EB staining detected with either gross blue staining or red fluorescence under a fluorescent microscope indicates the albumin extra-vasation, namely the opening of the BBB. Severe systemic inflammation does compromise the BBB and the compromised BBB can be revealed by EB staining [29,30]. In the present study, however, EB extra-vasation could not be found in any brain area (except non-BBB areas) 6, 12 or 24 h after the systemic LPS injection at all ages (P1, P3 and P7). This result primarily denied a breakdown of the BBB caused by low-dose systemic LPS. Moreover, transmission electron microscopy further proved that the capillaries remained intact 12 h after the systemic treatments of LPS in the P1 rats. The structure of the endothelial tight junctions was normal and the endothelial cells were intact without fenestration or endocytotic vesicles.

Although no morphological changes of the BBB were found in the present study, it is possible that the BBB permeability was increased by the peripheral treatment of LPS. In that case, the circulating molecules including cytokines and/or plasma proteins may cross the BBB and thus enter the brain parenchyma. Those increased peripheral molecules could in turn trigger some of the innate immune receptors, especially TLR4 that recognizes not only LPS but DAMPs [16]. As shown by real-time PCR, the mRNA expression of TLR4 was significantly up-regulated in the whole brain of the neonatal rats 12 h after the central injection of LPS, compared to the NS control. MyD88, the pivotal downstream signaling adaptor molecule recruited by the activated TLR4, leads to the expression of proinflammatory genes via NF-κB and mitogen-activated protein kinase (MAPK) signaling pathways [31,32]. The Western Blot assay showed that TLR4, MyD88, NF-κB p50 and TNF-α proteins were all significantly up-regulated 6, 12 and 24 h after the central injection of LPS in the P1 rat brain, indicating the activation of TLR4–MyD88 pathway. The immunohistochemical results showed that the microglial cells were strongly activated in the neonatal rat brain 12 h after icv. LPS injection. As a control, icv. NS injection did not induce microglial activation. These results suggest that the microglia in the brain and the TLR4–MyD88 innate immune pathway should be activated after direct contact with LPS in the neonatal rats, even at P1.

In this circumstance, that P1 CNS is able to respond to LPS by TLR4–MyD88 activation, the systemic injection of low-dose LPS did not induce the up-regulation of TLR4 protein in the neonatal rat brain (even in the P1 brain) at 6, 12 and 24 h after injection. Consistently, MyD88, NF-κB p50 or TNF-α was not induced to increase in these brains after the systemic injection of low-dose LPS. Moreover, our immunohistochemical observation showed resting microglial cells in the neonatal rat brains with the peripheral injection of LPS. These findings indicated that the innate immune reaction of the neonatal brain was not challenged by the systemic administration of low-dose LPS. As the dosage of systemic LPS administered in the present study was similar to that in LPS preconditioning, our data also suggest that the mechanism of LPS protection against HI may not be dependent on TLR4-mediated innate immune reaction in the neonatal brain.

## 3. Experimental Section

### 3.1. Animals and Experiment Procedure

Male and female neonatal Sprague-Dawley rats at P1, P3 and P7 were purchased from the Animal Center of the Fourth Military Medical University. For the present study, 205 rats were used. Rat pups were kept under standard conditions with a 12 h/12 h light/dark cycle. All experiments were done in accordance with the guidelines established by the Animal Care Committee of the Fourth Military Medical University and all efforts were made to minimize animal discomfort and to sacrifice the fewest animals (No. 14001, The Tab of Animal Experimental Ethical Inspection of the Fourth Military Medical University, 13 September 2013).

Either icv. or ip. administration of LPS (*Escherichia coli* lipopolysaccharide serotype 0111:B4, Sigma-Aldrich, St. Louis, MO, USA) were carried out for all the experiments in the present study. To perform icv. injections, the heads of rat pups were gently fixed, and a 10 µL microsyringe was inserted vertically into the central part of the right hemisphere, approximate 3 mm deep by the aid of the mark on the syringe tip. Three microliters of LPS (1 mg/mL) were injected slowly within 1 min, and the syringe was carefully withdrawn. The same volume of NS was used as control for all the LPS administration.

### 3.2. Evaluation of the BBB Permeability by EB

Male and female rats (P1, P3 and P7) were distributed randomly to LPS and pyrogen-free NS groups. The rat pups received ip. injection of LPS (0.01 mg/mL, 0.3 mg/kg·bw) or the same volume of NS as the control. Then the animals returned to a warm and humid incubator and were fed with milk every 3 h. At 6, 12 or 24 h after LPS or NS, the pups were anesthetized with ip. injection of pentobarbital sodium (50–60 mg/kg·bw), followed by an intracardiac injection of 2% EB (4 mL/kg·bw, Sigma-Aldrich) dissolved in 0.9% NaCl solution. One minute later, the pups were perfused transcardiacally with 20 mL NS to flush the blood away within 2–3 min, and the brains were removed and sectioned at 25 μm thickness with a cryostat to investigate EB albumin extravasation using an Olympus BX 51 microscope (Olympus, Tokyo, Japan).

### 3.3. Electron Microscopy

The P1 neonatal rats (*n* = 2 for each treatment) were anesthetized 12 h after ip. injections of LPS (0.01 mg/mL, 0.3 mg/kg·bw) or the same volume of NS. Then they were transcardially perfused with 4% polyformaldehyde–0.025% glutaraldehyde in 0.1 M phosphate buffer (pH 7.4). The brains were then removed and cut using a vibratome. After dehydration in graded alcohol and 1,2-epoxypropane, the sections were embedded in Epon 812. Representative brain areas such as the frontal cortex and hippocampus were chosen for ultrathin sectioning. The ultrathin sections were mounted onto 200 mesh copper grids, lightly stained with uranium acetate and lead citrate, and examined with a transmission electron microscope (JEM-100SX, JEOL, Tokyo, Japan). For each brain area, such as cortex or hippocampus, at least three chosen fields were scanned, the fine structure of the endothelial cells, particularly tight junctions and endocytotic vesicles were investigated.

### 3.4. Western Blot Assay

The P1 rats were divided randomly into 4 groups (*n* = 4 for each time point/per group) including ip. injections of LPS (0.01 mg/mL, 0.3 mg/kg·bw) or the same volume of NS, icv. injection of LPS (1 mg/mL, 3 µL) or the same volume of NS. The animals were anesthetized and killed by decapitation 6, 12 or 24 h after injection. The brains were removed on ice and immersed in liquid nitrogen within 2 min to prevent protein degradation. Briefly, brain samples (about 0.05g) were homogenized with 0.5 mL of ice-cold Radio-Immunoprecipitation Assay (RIPA) lysis buffer (20 mM Tris–HCl, pH 7.5, 1 mM ethylene diamine tetraacetic acid (EDTA), 5 mM MgCl_2_, 1 mM d,l-dithiothreitol (DTT), 20 μg/mL aprotinin, 1 mM Phenylmethanesulfonyl fluoride (PMSF) and 2 mM sodium orthovanadate). The homogenates were centrifuged at 13,000 rpm at 4 °C for 10 min and the deposit was removed. The protein concentration was determined using Bradford method, a detergent-compatible protein assay with a bovine serum albumin as standard. Protein samples were boiled with loading buffer at 100 °C for 10 min, electrophoresed on 10% sodium dodecyl sulfate-polyacrylamide gel electrophoresis (SDS-PAGE) and transferred onto a nitrocellulose membrane (Millipore, Bedford, MA, USA). The filter membranes were blocked with 5% nonfat milk at 37 °C for 1 h and incubated with different primary antibodies (TLR4, 1:500, Abgent, San Diego, CA, USA; MyD88, 1:500, Cell Signaling Technology, Boston, MA, USA; NF-κB, 1:2000, Epitomics, Burlingame, CA, USA; TNFα, 1:500, Sigma-Aldrich, St. Louis, MO, USA; β-actin, 1:2000, Cwbio, Beijing, China).

The P3 and P7 neonatal rats were randomly divided into 4 groups, respectively, as mentioned above. Twelve hours after injection, the animals were anesthetized and killed. The brains were removed and Western Blot was performed. The expressions of MyD88 and NF-κB p50 were detected.

### 3.5. Quantitative Real-Time Reverse-Transcriptase (PCR) Analysis

TLR4 mRNA within the whole brains was detected by quantitative real-time PCR. Male and female neonatal rats (P1, P3 and P7, *n* = 4 for each time point/per group) were divided randomly into 4 groups as mentioned above. Twelve h after injection, animals were anesthetized and killed by decapitation. The brains were removed on ice and immersed in liquid nitrogen within 2 min to prevent RNA degradation. Total RNA of the whole brain was extracted and homogenized in RNAiso Plus (Code No: 9108, TaKaRa, Dalian, China) according to the manufacturer’s instructions. Every container of the RNA samples was pre-treated with diethylpyrocarbonate (DEPC). RNA samples were treated using RNase-free DNaseI (TaKaRa) to remove any traces of contaminating DNA. One mg of total RNA was used as a template to make first strand cDNA in each 20 μL reaction mixture, according to the manufacturer’s instructions (TaKaRa). Primers used were designed and synthesized by a company (Haining bio Co, Ltd., Xi’an, China) as follows: TLR4 (NM_019178.1), sense: 5'-GGCATCATCTTCA TTGTCCTTG-3', and antisense: 5'-AGCATTGTCCTCCCACTCG-3'; the expected size was 111 bp; β-actin (NM_031144), sense: 5'-GGAGATTACTGCCCTGG CTCCTA-3', and antisense: 5'-GACTCATCGTACTCCTGCTTGCTG-3'; and the expected size was 150 bp. Quantitative analysis was performed by monitoring in real time the increase in fluorescence of the SYBR green dye on CFX96™ Real-Time PCR Detection System (Bio-Rad, Hercules, CA, USA). The PCR mixture (20 μL) contained 1 μL of each primer, 2 μL purified cDNA template (less than 100 ng cDNA), 6 μL RNA-enzyme free distilled water and 10 μL SYBR^®^ Premix Ex Taq™ II (TaKaRa, Dalian, China). The procedure was performed according to the manufacturer’s instructions. Denaturing and annealing times were 10 and 30 s each, at 95 and 58 °C, respectively. cDNA samples were amplified for 40 cycles and melt curves were investigated. Delta method was used for fold changes of gene expression. The results were analyzed by the system of CFX Manager 2.1 (Bio-Rad, Hercules, CA, USA).

### 3.6. Immunohistochemical Analysis

The P1, P3 and P7 neonatal rats were distributed randomly to 4 groups (*n* = 3 for each group) as mentioned above. Twelve hours after injection, the animals were anesthetized and killed. The whole brains were carefully dissected out and immersed in 4% paraformaldehyde solution. Forty-eight hours later, the brains were transferred into 20% sucrose solution at 4 °C for 24–48 h until they sank. Coronary sections were cut at 20 μm in thickness with a cryostat and thawed-mounted onto gelatinized slides. The sections were kept at −20 °C until used.

Five different coronal sections were chosen in each rat. The sections were rinsed in phosphate buffered saline (PBS) three times (5 min each time) and incubated with primary antibodies for 16–24 h at room temperature. Primary antibodies were Iba-1 (1:1000, rabbit anti rat, Wako, Tokyo, Japan) and RECA 1 (1:400, mouse anti rat, Abcam, Cambridge, UK). Then the sections were rinsed with PBS for 3 times and incubated with the appropriate secondary antibodies (1:800, anti mouse IgG, Alexa Fluor 594, Invitrogen, Grand Island , NY, USA; 1:800, anti-rabbit IgG, Alexa Fluor 488, Invitrogen, Grand Island, NY, USA) for 4 h at room temperature. The sections were rinsed with PBS 3 times again, incubated with Hochest33342 (1:10,000, Sigma-Aldrich, St. Louis, MO, USA;) for 10 min, rinsed with PBS for 3 times and examined on a FV 1000 confocal microscope (Olympus, Tokyo, Japan).

### 3.7. Statistical Analysis

All data were presented as mean ± standard deviation. The statistical significance of differences between groups was determined by one-way analysis of variance. The statistical program SPSS 19.0 for windows (IBM SPSS, Chicago, IL, USA) was used for statistical analysis and the significance was considered at *p* ≤ 0.05.

## 4. Conclusions

In conclusion, our study demonstrates that: (1) microglia and TLR4-MyD88 pathway-associated innate immune reactions can be activated in the brain after direct contact with LPS in neonatal rats; (2) systemic low-dose LPS may not be able to induce a transient opening or breakdown of the immature BBB even in P1 rats; and (3) systemic low-dose LPS can activate neither the microglia nor the TLR4–MyD88 pathway in neonatal rat brain. Taken together, our data imply that the CNS of neonatal rats possess complete innate immune systems, including resistant BBBs and responsive TLR4-mediated reactivity to defend against bacterial endotoxin, and that low-dose LPS, systemically administered, does not compromise the BBB or induce TLR4-mediated innate immune reaction in the neonatal brain.
